# Small intestinal development in suckling rats after enteral obestatin administration

**DOI:** 10.1371/journal.pone.0205994

**Published:** 2018-10-19

**Authors:** Monika Słupecka-Ziemilska, Paulina Grzesiak, Michał Jank, Alicja Majewska, Agnieszka Rak, Paweł Kowalczyk, Ikuo Kato, Atsukazu Kuwahara, Jarosław Woliński

**Affiliations:** 1 Department of Animal Physiology, The Kielanowski Institute of Animal Physiology and Nutrition, Polish Academy of Sciences, Jabłonna, Poland; 2 Division of Pharmacology and Toxicology, Department of Pre-Clinical Sciences, Faculty of Veterinary Sciences, Warsaw University of Life Sciences SGGW-WULS, Warsaw, Poland; 3 Department of Physiological Sciences, Faculty of Veterinary Sciences, Warsaw University of Life Sciences SGGW-WULS, Warsaw, Poland; 4 Department of Physiology and Toxicology of Reproduction, Institute of Zoology and Biomedical Research, Jagiellonian University in Krakow, Krakow, Poland; 5 Department of Animal Nutrition, The Kielanowski Institute of Animal Physiology and Nutrition, Polish Academy of Sciences, Jabłonna, Poland; 6 Department of Medical Biochemistry, Kobe Pharmaceutical University, Kobe, Japan; 7 Laboratory of Physiology, Institute for Environmental Sciences and Graduate School of Nutritional and Environmental Science, University of Shizuoka, Shizuoka, Japan; Chuo University, JAPAN

## Abstract

This study investigated the effect of enteral administration of obestatin on the development of small intestine, as well as oxidative stress markers and trancriptomic profile of gastrointestinal genes. Suckling rats were assigned to 3 groups treated with: C-saline solution; OL-obestatin (125 nmol/kg BW); OH-obestatin (250 nmol/kg BW) administered twice daily, from the 14^th^ to the 21^st^ day of life. Enteral administration of obestatin in both studied doses had no effect neither on the body weight of animals nor the BMI calculated in the day of euthanasia. Compared to the control group (C), treatment with obestatin resulted in significant changes in the histometry of the small intestinal wall as well as intestinal epithelial cell remodeling. The observed changes and their possible implications for intestinal development were dependent on the dosage of peptide. The enteral administration of high dose (OH) of obestatin significantly decreased its expression in the stomach and increased markers of oxidative stress. The gene profile revealed MAPK3 (mitogen-activated protein kinase-3) as the key regulator gene for obestatin action in the gastrointestinal track. In conclusion, we have showed that enteral administration of obestatin influences the gut mucosa remodeling. It is also suggested that the administration of high dose (OH) has inhibitory effect on the intestinal maturation of suckling rats.

## Introduction

Obestatin, a 23-amino acid peptide, was originally purified from the rat stomach as a ghrelin-accompanying peptide, created during the posttranslational modification of polypeptide precursor of ghrelin (preproghrelin) [1]. Currently, G protein-coupled receptor 39 [1] and glucagon-like peptide 1 receptor [2] are debatable targets of obestatin. In the study by [1] it was reported that obestatin shows opposite biological effects to ghrelin and inhibits food intake, body weight gain, gastric emptying and jejunal contractility. However, the last decade investigations on the physiological role of obestatin demonstrate that obestatin is an independent peptide, participating in many physiological processes in the organism including the development of the gastrointestinal tract in the early postnatal period. Previous studies of our team have shown that obestatin influences small intestinal contractility in rats [3,4]. We found that obestatin effect is dependent on the age of animals, segment of jejunum and route of peptide administration (*in vitro* studies with obestatin in the intestinal luminal environment *vs*. enteral administration to suckling animals). Moreover, the presence of obestatin has been reported in colostrum and milk [5–7], which firmly proves the significance of obestatin (both endogenous and exogenous) in the regulation of gastrointestinal function in the early postnatal period. It is also worth mentioning the study, in which the expression of obestatin immunoreactive (IR) cells in the both stomach mucosa and myenteric plexus of neonatal rats starting from the 1^st^ day after birth was observed. It was shown that obestatin IR cells in the stomach mucosa reach the same number as in adults by 6 weeks after birth, whereas ghrelin-IR cells were not observed on the 1^st^ day of life. Although their number increased within first week and remained at this plateau until the month of life, the expression of ghrelin-IR cells were still lower than those of the obestatin-IR cells [8]. The results show that in the stomach mucosa, obestatin-IR cells are identified mainly in the fundus and, to some extent, also in the corpus and antrum. Therefore, the aims of the study were to understand how obestatin administrated enterally effects on the structural and functional development of intestines in suckling ratsand at the same time to assess (via the indication of the activity of glycosylases—markers of oxidative stress) the potential side effect of this supplementation on the small intestine samples.

## Material and methods

All the experimental procedures involving animals were conducted in compliance with the European Union regulations concerning the protection of experimental animals (EC Directive 86/609/EEC with amendments). The study protocol was approved by the 3^rd^ Local Ethics Committee in Warsaw, according to the Polish Law for the Care and Use of Animals (Resolution no 50/2012).

### Obestatin

Rat obestatin was synthesized at the Yanaihara Institute using a solid phase method with an Fmoc-strategy and an automated peptide synthesizer (Applied Biosystem 9030 Pioneer, Foster, CA, USA). Analytical HPLC and MALDI-TOF MS confirmed the homology of the product. The hormone was kept in powder form at -20°C and then dissolved in saline solution (0.9% NaCl) to the final concentration, just before use.

### Animals

At the start of the experiment 12 male and 12 female Wistar Han rats (13 weeks old) were used. Animals were purchased from the Center of Experimental Medicine at the Medical University of Bialystok. After two weeks of acclimatization the rats were mated. After mating, females were separated from males and they were allowed free access to commercial rat breeding chow (5% fat; 3.1 kcal/g, Wytwornia Pasz Morawski, Poland) and tap water in a humidity- and temperature-controlled room on a 12-h:12-h light:dark cycle. Twenty-four hours after delivery litters were standardized to 10 pups. On the 14^th^ day of life the rat pups (n = 120) form 12 litters were randomly assigned to one of 3 treatment groups (4 litters for each treatment): C- control animals- treated with saline solution; OL- pups treated with a lower dose of obestatin at 125 nmol/kg BW; OH-pups treated with a high dose of obestatin at a 250 nmol/kg BW. Both pharmacological doses of obestatin were constructed based on the previous *in vitro* studies on intestinal contractility in rats [3,4]. Prior to euthanasia, all pups were housed with their mothers and breast-fed *ad libitum*. Saline solution or obestatin were administered enterally via oral gavage twice a day staring from the 14^th^ day of life until the 21^st^ day of life. On the 21^st^ day of life (30 min after the morning NaCl/obestatin treatment) 4 randomly selected rat pups from each litter (n = 16 for each treatment) were euthanized by CO_2_, while the rest were taken for another experiment.

Duodenum, middle jejunum and ileum were transected and samples of each section were collected and immediately fixed in a 10% neutral formalin solution or deep frozen and stored in -80 °C. Blood for radioimmunoassays was collected via cardiac puncture, withdrawn on EDTA and aprotinin (0.6 TIU/mL of blood) than immediately centrifuged (1.600 x g, 15 min, 4 °C), distributed to Eppendorf tubes, deep frozen and stored until analysis (-80 °C).

### Microarray analysis

Tissue samples were homogenized using TissueLyser LT (Qiagen, USA). Total RNA from homogenized tissue samples was isolated using Rneasy Mini Kit (Qiagen, USA). DNAse I digestion as a step of isolation protocol was added to remove contamination of sample with DNA. This was performed using RNase-Free DNase Set (Qiagen, USA). NanoDrop (NanoDrop Technologies, USA) was used for quantification of RNA. Final RNA quality and integrity were analysed using Agilent 2100 Bioanalyzer (USA) with the use of RNA 6000 Nano Kit (Agilent, Germany). Only RNA samples with Relative Integrity Number (RIN) number ≥7.8 were considered as optimal and included in further analysis.

Gene expression profile was evaluated using Agilent platform, including Agilent-028279 SurePrint G3 Rat GE 8x60K Microarray (Agilent Technologies, USA) and Agilent Technologies Reagent Set. All procedures were run according to the manufacturer’s protocols. Internal control, added to the samples, came from RNA Spike In Kit (Agilent Technologies, USA), whereas amplification and labelling of target DNA in order to obtain complementary RNA (cRNA) were performed using Low Input Quick Amp Labeling Kit. Fragmentation and hybridization of cRNA were performed using Gene Expression Hybridization Kit and slides after hybridization were washed using Gene Expression Wash Buffer Kit. Agilent DNA Microarray Scanner G2505C was used for acquisition and analysis of hybridization intensities.

For the microarray experiment tissues from 4 randomly selected rats per one experimental group were sampled, representing both stomach and middle part of intestine; control group (C) or High Obestatin (OH) groups. For the purposes of microarray experiment we were using common reference design, in which all the studied samples were hybridized against the pool of equal amounts of RNA originating from all rats participating in the study. The cRNA of common reference was labelled with Cy3 and the cRNA obtained from investigated tissues were labelled with Cy5. Sixteen two-colour microarrays were performed, each representing specific rat (n = 4 microarrays with stomach samples from C group; n = 4 microarrays with middle intestine samples from C group; n = 4 microarrays with stomach samples from NOH group and n = 4 microarrays with middle intestine samples from NOH group). The equal amounts of cRNA samples (300ng) were hybridized on each slide. Since the microarrays were dual-colour on each slide we hybridized one Cy3-labelled common reference sample and one Cy5-labelled experimental rat sample. Once the microarrays were scanned after hybridization and washing they were analysed in Agilent Feature Extraction (FE) Software version 10.7.3.1. FE. This software extracts the data and performs its background subtraction as well as performs Lowess normalization of obtained intensities.

The probes intensities were than downloaded to PANTHER Version 13.0 software (Protein ANalysis THrough Evolutionary Relationships) and Pathway Studio 11.4.0.8 software (Ariadne Genomics) in order to perform ontologic analyses, identify differentially regulated signalling pathways and function of differentially regulated genes.

#### Verification of results by real-time PCR

The expression of two genes (*MAP2K3 and MAPK3)* was verified using real-time PCR method. *GAPDH* was used as a reference gene. Transcription of RNA to cDNA was done using High-Capacity cDNA Reverse Transcription Kit (Applied Biosystems) and Real time PCR reaction was performed in triplicate using a Brilliant III Ultra-Fast SYBR Green QPCR Master Mix kit (Agilent Technologies, USA) on Stratagene Mx3005P Quantitative PCR instrument according to the manufacturer’s protocol. Conditions of real time PCR reactions were according to the manufacturer’s protocol and comprised the following steps: polymerase activation at 95°C for 3 minutes; 40 cycles of amplification (95°C for 10 seconds, 60°C for 20 second). The primers were designed using Primer-Blast software (NCBI database) and verified using Oligo Calc: Oligonucleotide Properties Calculator (free software available online, provided by Northwestern University) to exclude sequences showing self-complementarity. The sequences of primers were given in Table 1.

**Table 1:** Primers sequences used for real-time PCR.

Gene	Forward primer (5’-3’)	Revers primer (5’-3’)	NCBI accession number
*MAP2K3*	*CCATTCTGCGATTCCCTTAC*	*GCAATGTCCGTCTTCTTAGT*	NM_001100674
*MAPK3*	*GTCATAGGCATCCGAGACA*	*CGCAGGTGGTGTTGATAAG*	NM_017347
*GAPDH*	*CTCTGCTCCTCCCTGTTCTA*	*CAATGAAGGGGTCGTTGATG*	NM_017008.4

The results were calculated using 2^- ΔΔCt^ method [9]. The expression changes were determined as relative expression of genes and normalized to reference gene *GAPDH*. The results are presented as log_2_.

### Sample preparation for histomorphometry, proliferation and apoptosis rate

The preparation of the intestinal samples for the histomorphometry of the intestinal wall and the assessment of the proliferation activity and apoptosis in the intestinal epithelium was performed on paraffin embedded sections and described in detail by Słupecka et al. [10]. Briefly, for intestinal histometry studies, slides were stained with hematoxylin and eosin. The villi lengths, crypt depth, thickness of mucosal layer (from the tip of the villi to the bottom of the crypt) were visualized using a light microscope (Axioskop 40, Zeiss, Germany) and analyzed using Axio Vision 4.2 Release software (Zeiss, Germany).

The number of apoptotic epithelial cells was determined using a TUNEL assay (terminal deoxynucleotidyl transferase-mediated dUTP nick end labeling). The procedure was performed according to the manufacturer’s protocol for the assay (ApopTag, Merck Millipore, Darmstadt, Germany USA). Slides were visualized using a microscope under 400x magnification. The apoptotic index was estimated separately for crypt and villi area. The apoptotic index was expressed as the number of apoptotic cells on all epithelial cells of villi and crypt respectively. Proliferating crypt cells were immunostained for rabbit polyclonal anti- Ki67 antibodies (Abcam, UK), 50 times diluted in 1% BSA-PBS and processed according to manufacturer’s protocol for EnVision+system (DakoCytomation, Denmark), providing secondary antibodies conjugated with HRP enzyme and the chromogen (diaminobenzidine) as the substrate for HRP enzyme. The slides were counterstained with hematoxylin. The mitotic index was calculated as the number of Ki67 positive cells on all epithelial cells of the crypt cross-section. For all abovementioned analysis a minimum of 15 well-oriented and intact villi and crypts were randomly selected per slide by an investigator blinded to treatment allocation.

### Immunofluorescence of obestatin

The immunofluorescence staining procedure was carried out according to the method described in our previous publication [10]. After stomach samples were fixed in 10% neutral formalin solution and rehydrated, they were incubated with the primary antibody against obestatin in the block buffer (1% BSA-PBS), 200 times diluted then incubated with secondary antibody conjugated with FITC (Abcam, UK). Both incubations were done at room temperature for 1h. After being rinsed 3 times, the slides were mounted, and visualized through a confocal microscope (model LSM 5 Pascal, Zeiss, Germany).

### Western blotting

Tissue preparation, lysis, Western blotting and quantification were performed as a standard procedure and were described previously [11]. In brief, the samples were separated by 15% SDS-PAGE (Mini-Protean II Electrophoresis Cell, BioRad). 60 μg of proteins were transferred to PVDF membranes (Merck Millipore, Darmstadt, Germany) and incubated with anti obestatin antibody (Abcam, Great Britain) diluted to 1:200 at 4°C overnight. Then, the membranes were incubated with a horseradish peroxidase-conjugated antibody (Santa Cruz Biotechnology, CA, USA) diluted to 1:500. Signals were detected by chemiluminescence using the Western blotting luminol reagent and visualized using a ChemiDoc-It Imaging System (UVP, LLC. Cambridge, UK). All bands visualized by chemiluminescence were quantified using Image J analysis software (US National Institutes of Health, Bethesda, MD, USA). The blots were then stripped and probed for anti-β-actin.

### Brush border enzyme activity

Brush border enzyme activity was measured in the mucosa scrapings from the middle part of the jejunum. The mucosa was scraped off with a microscope slide and deep-frozen in liquid nitrogen (–80 °C). After thawing, the sample was homogenized in cold distilled water (1 g intestinal mucosa/5 ml distilled water) and centrifuged for 5 min at 1000 *g* at 4 °C. The protein content was then determined as described by Hartree [12], using BSA as the standard. The activities of aminopeptidase A and N were assayed with N̈-glutamyl- *p*-nitroanilide and N̈-leucyl-*p*-nitroanilide as substrates respectively [13] and that of dipeptidyl peptidase IV was assayed with glycyl-N̈-prolyl-*p*-nitroanilide [14]. The resulting enzymatic units (IU) are expressed as μmol *p*-nitroanilide released/min at 37 °C. Lactase, maltase and sucrase were determined as described earlier [15] with minor modifications.

### Markers of oxidative stress

The oxidative stress in the small intestinal tissue was investigated by the measurement of DNA repair activity and gene expression of Cox-2.

#### DNA repair activity by nicking assay

The 1 cm length samples of middle jejunum were homogenized with 4 volumes of 50 mM Tris—HCl, pH 7.5, buffer containing 1 mM EDTA and protease inhibitor cocktail (Sigma Aldrich, Germany). Cells were disrupted by sonication (three 15-s pulses with 30-s intervals). The cell debris was removed by centrifugation (7000 g, 4 °C, 15 min), and the supernatant used as the source of DNA repair enzymes (alkyl-N-purine-DNA (ANPG) and 8-oxoguanine DNA (OGG1) glycosylazes) was collected. Protein concentration was determined by the Bradford method [16]. Supernatants were stored in aliquots at −80 °C for further analysis. Etheno adduct excision activity was measured by the nicking assay using an oligodeoxynucleotide duplex containing a single 8oxoG (for OGG1 activity) and ethenoA residue (for ANPG activity) according to Speina et al. [17] and Obtułowicz et al. [18].

#### Gene expression of COX-2

The gene expression of Cox-2 was measured by Real Time RT-PCR method [19–21]. Briefly, total RNA was extracted from rat tissue using the PureLink RNA kit (Invitrogen, USA). RNA quantity and purity were determined using NanoDrop microspectrophotometer ND-1000 (Wilmington, DE). One μg of total RNA was reverse-transcribed to high capacity cDNA using a reverse transcription kit including mixture of random primers (Applied Biosystems, Foster City, CA). Real time quantitative RT-PCR analysis was carried out by SYBR Green I dye detection (Applied Biosystems, Foster City, CA) using the real time detection system (Bio-Rad). Real-time PCR primers were designed with PrimerExpress software was as follows:

Cox-2 F, GATTGACAGCCCACCAACTT Exon 4, NM_017232
Cox-2 R, CGGGATGAACTCTCTCCTCA Exon 5

Primers were synthesized in DNA Sequencing and Oligonucleotide Synthesis Laboratory IBB, PAS (Poland). Optimized conditions were as follows: 1 min at 48 °C, 7 min, and 30 s at 95 °C and 45 cycles of 20 s at 95 °C and 35 s at 62 °C in which an optical acquirement were performed. Melt curves were performed upon completion of the cycles to ensure absence of nonspecific products. Quantification was performed by normalizing cycle threshold (*Ct*) values with the Cox2 control gene 18s, and analysis was carried out with the 2−ΔΔ*CT* method [9].

### Obestatin and ghrelin radioimmunoassay

Rat plasma samples were assayed for obestatin and total ghrelin (acyl and des-acyl) concentration using commercially available radioimmunoassay (RIA) kits: Ghrelin (Rat, Mouse) RIA Kit and Obestatin (Rat, Mouse) Ultra-Sensitive RIA Kit (Phoenix Pharmaceuticals, Inc., USA) according to the manufacturer’s instructions. Spike and recovery of obestatin and total ghrelin were measured in three different plasma samples. The assays were performed in one run. The mean percent recovery for obestatin and ghrelin in plasma was 90% and 93%, respectively. The intra-assay coefficient of variation (CV) for obestatin and ghrelin in plasma was 5.7 and 6.5%, respectively.

### Statistics

The statistical analysis of microarrays was performed using Gene Spring 14 software (Agilent, USA). The probe sets were filtering by flags to remove poor quality probes (absent flags). The statistical significance of the differences was evaluated using a one-way ANOVA and Tukey’s HSD Post-hoc test or Kruskal-Wallis test (p < 0.05). A multiple testing correction was performed using Benjamini and Hochberg False Discovery Rate (FDR) < 0.05. Microarray data were deposited at the Gene Expression Omnibus data repository under the number GSE102217. The rest data obtained in the study were analyzed using an Unpaired t-test, Mann-Whitney test, one-way ANOVA and Tukey’s post-hoc test or Kruskal-Wallis test (p < 0.05) (Prism 6 for Mac OS X, Version 6.0h, GraphPad Software, Inc., USA).

## Results

### Obestatin effect on body weight, adiposity and intestinal morphology

Enteral administration of obestatin in both studied doses had no effect neither on the body weight of animals nor the BMI calculated in the day of euthanasia. Adiposity estimated either by the weight of peritoneal fat pad did not differ between studied groups. No differences were found in the weight and length of the intestine between studied groups (Table 2).

**Table 2:** Obestatin effect on body weight, adiposity and intestinal morphology in suckling rats.

Parameter / Group	C	OL	OH	*P*
Body weight (g)	36.9 ± 1.6	35.7 ± 1.0	36.0 ± 1.4	*0*.*8286*
BMI (kg/m^2^)	3.18 ± 0.07	3.24 ± 0.07	3.14 ± 0.08	*0*.*6120*
Peritoneal fat (g/ kg BW)	0.039 ± 0.006	0.039 ± 0.005	0.035 ± 0.005	*0*.*8446*
Small intestine weight (g/kg BW)	0.044 ± 0.001	0.046 ± 0.0009	0.046 ± 0.001	*0*.*4922*
Small intestine length (cm)	45.9 ± 1.1	47.4 ± 1.3	46.0 ± 1.2	*0*.*6470*

C—saline solution (twice a day); OL—intra stomach obestatin (125 nmol/kg BW, twice a day); OH—intra stomach obestatin (250 nmol/kg BW, twice a day); values are given as means ± SEM.

### Obestatin effect on gene expression profile

**Fig 1:**
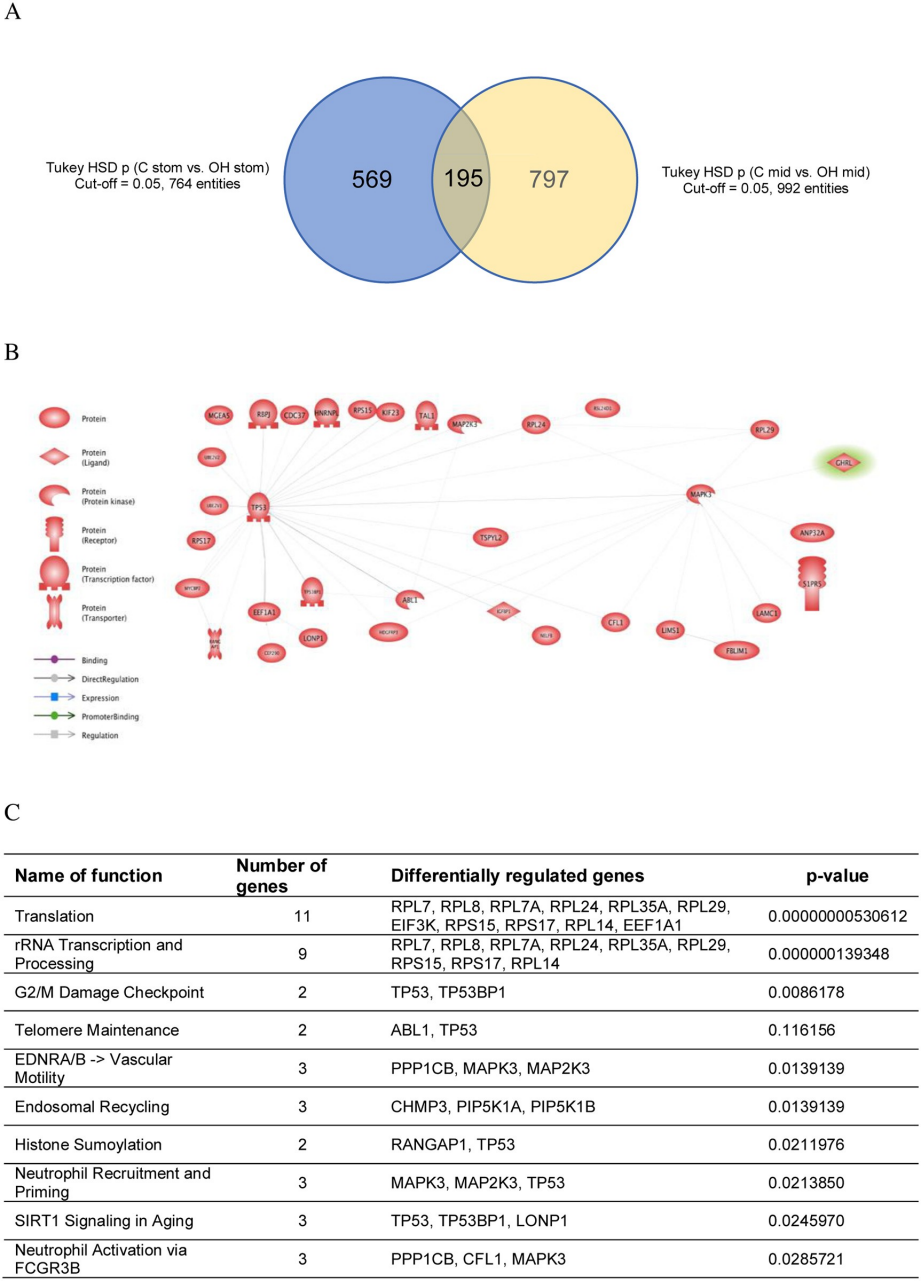
Integrative analysis of microarray data identifying significantly changed genes. (A) Venn diagram of overlapping genes which expression significantly changed in the stomach (blue) and intestine (yellow) after enteral administration of obestatin in dose 250 ug/kg BW to suckling rats (group OH). (B) The analysis of direct relations between differentially regulated genes. (C) Results of Gene Ontology analysis.

Analysis started from identification of all differentially expressed (DE) genes between two treatments in specific parts of gastrointestinal tract using Tukey’s HSD Post-hoc test. The number of DE genes in stomach was 764 whereas in middle part of intestine was 992. In order to identify the genes regulated by obestatin treatment the intersection of these two data sets was created. It revealed 195 differentially expressed probes. Out of this group it was possible to identify 131 transcripts representing specific genes (Fig 1A).

The analysis of Gene Ontology of this data set revealed significant regulation of genes involved in biological functions (Fig 1C). The analysis of direct relations between differentially regulated genes revealed that if we consider obestatin direct action on specific genes expression the key regulated genes are *MAP2K3* (coding mitogen- activated protein kinase kinase 3) and *MAPK*3 (coding mitogen-activated protein kinase-3) which regulates expression of other differentially regulated genes included tracriptional factor p53 with the crucial role in the cell response to DNA damage (Fig 1B).

**Fig 2:**
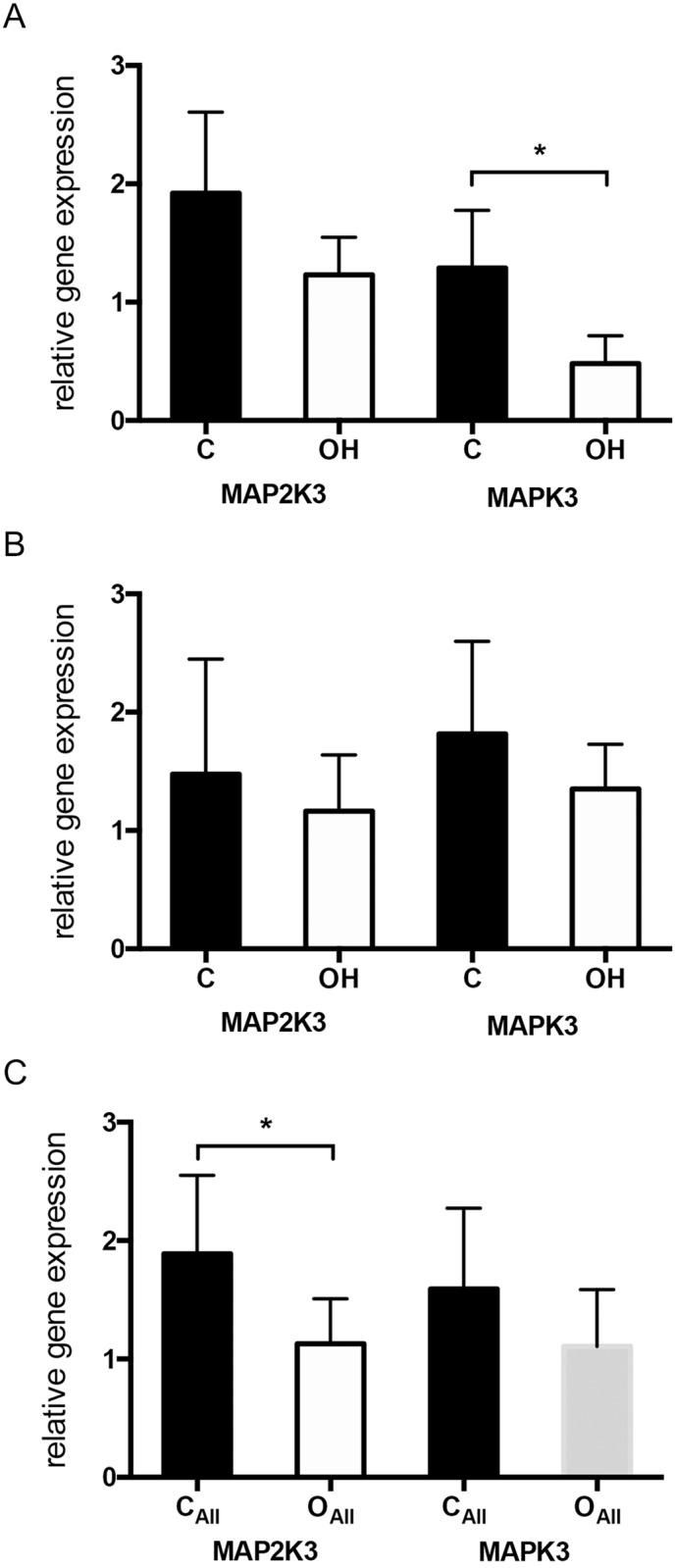
The relative expression of *MAP2K3* and *MAPK3* mRNA measured with real-time PCR in extracts from rats gastrointestinal tract. (A) Genes expression in the stomach of suckling rats administered with saline solution (group C) and after enteral administration of obestatin in dose 250 ug/kg BW (group OH); * p = 0.0314. (B) Genes expression in the intestine of suckling rats administered with saline solution (group C) and after enteral administration of obestatin in dose 250 ug/kg BW (group OH). (C) Genes expression in the gastrointestinal tissues (stomach+ intestine) of suckling rats administered with saline solution (group C_ALL_) and animals treated with obestatin in dose 250 ug/kg BW (group OH_ALL_); * p = 0.312. Values are given as means ± SEM.

To validate the microarray data the expression of *MAP2K3* and *MAPK3* was analyzed using real-time qPCR. The results showed significantly decreased expression of *MAPK3* in the stomach of rats treated with obestatin (OH) in comparison to control rats (C), (Fig 2A). Administration of obestatin in a high dose (OH) had no statistically signicficant effect on *MAP2K3* and *MAPK3* expression in the intestine (Fig 2B). However, when the stomach and intestine samples od rats treated with obestatin were analyzed together as one group OH_ALL_ (to show obestatin effect in gastrointestinal track) we observed significant decrease in the expression of *MAP2K3* in comparison to tissues (stomach and intestine) not treated with the peptide (group C_ALL_), (Fig 2C).

### The effect of obestatin administration on its blood plasma concentration and peptide expression and localization in the stomach

**Fig 3:**
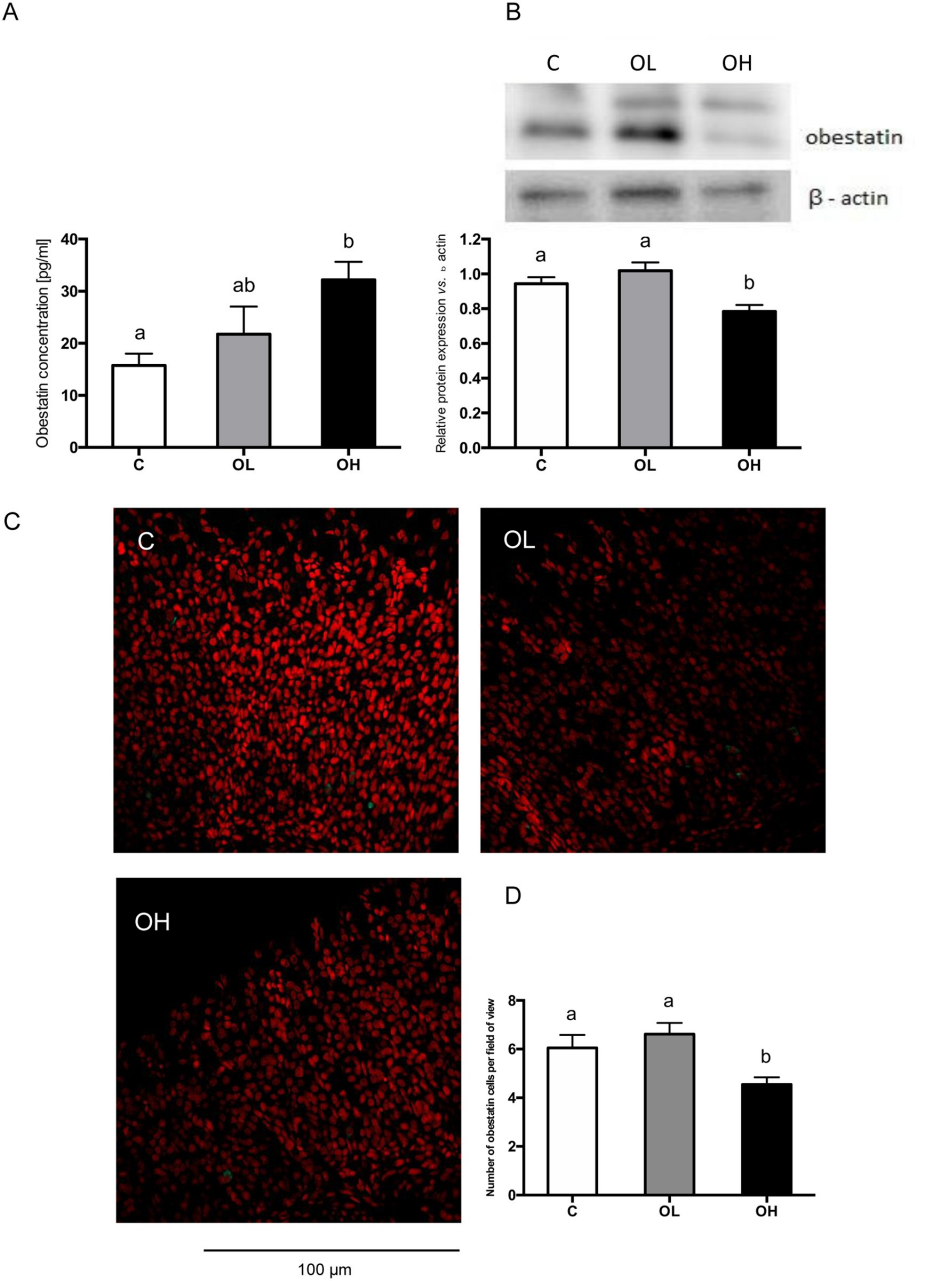
Obestatin effect on its concentration in plasma (A), peptide expression (B, D) and localization (C) in suckling rats. A) Radioimmunological assessment of obestatin concentration (pg/ml) in blood plasma. (B) Western blot analysis of obestatin expression in the middle jejunum segments in suckling rats. Results are presented as the optical density of the obestatin b-actin ratio in the study groups, performed in 6 replications. (C) Obestatin expression on confocal images in representative cross-sections from the stomach fundus of suckling rats. Obestatin cells are marked by green fluorescence. Nuclei were stained with 7-AAD (red fluorescence), (400x). (D) Mean number of obestatin cells in the oxyntic mucosa of stomach fundus per field of view (400x), n = 6. C—animals administered with saline solution (twice a day); OL—intra stomach obestatin (125 nmol/kg BW, twice a day); OH—intra stomach obestatin (250 nmol/kg BW, twice a day); values are given as means ± SEM; different superscript letters indicate statistical differences between the groups; p≤ 0.05.

In comparison with that in suckling rats administered saline solution, the obestatin concentration in blood plasma of suckling rats administered with obestatin was significantly increased (p = 0.0061) in the group treated with the high dose (OH), (Fig 3A).

Western blot and immunohistochemical analyzes detected that enteral obestatin administration to suckling rats influences on its peptide expression in the stomach (Fig 3B and 3C). Administration of the lower dose of obestatin (OL) significantly increased both the peptide expression in the stomach (Fig 3B) and the percentage of cells containing visible antigen in comparison to control animals (C), (p = 0.0012 and p = 0.0016, respectively) (Fig 3D). High dose of obestatin (OH) gave the opposite effect, a significant decrease in comparison to C group. Immunohitochemistry revealed obestatin localization in the oxyntic gland of gastric mucosa predominantly in the base and neck area (Fig 3C).

### Obestatin effect on intestinal epithelium renewal

**Fig 4:**
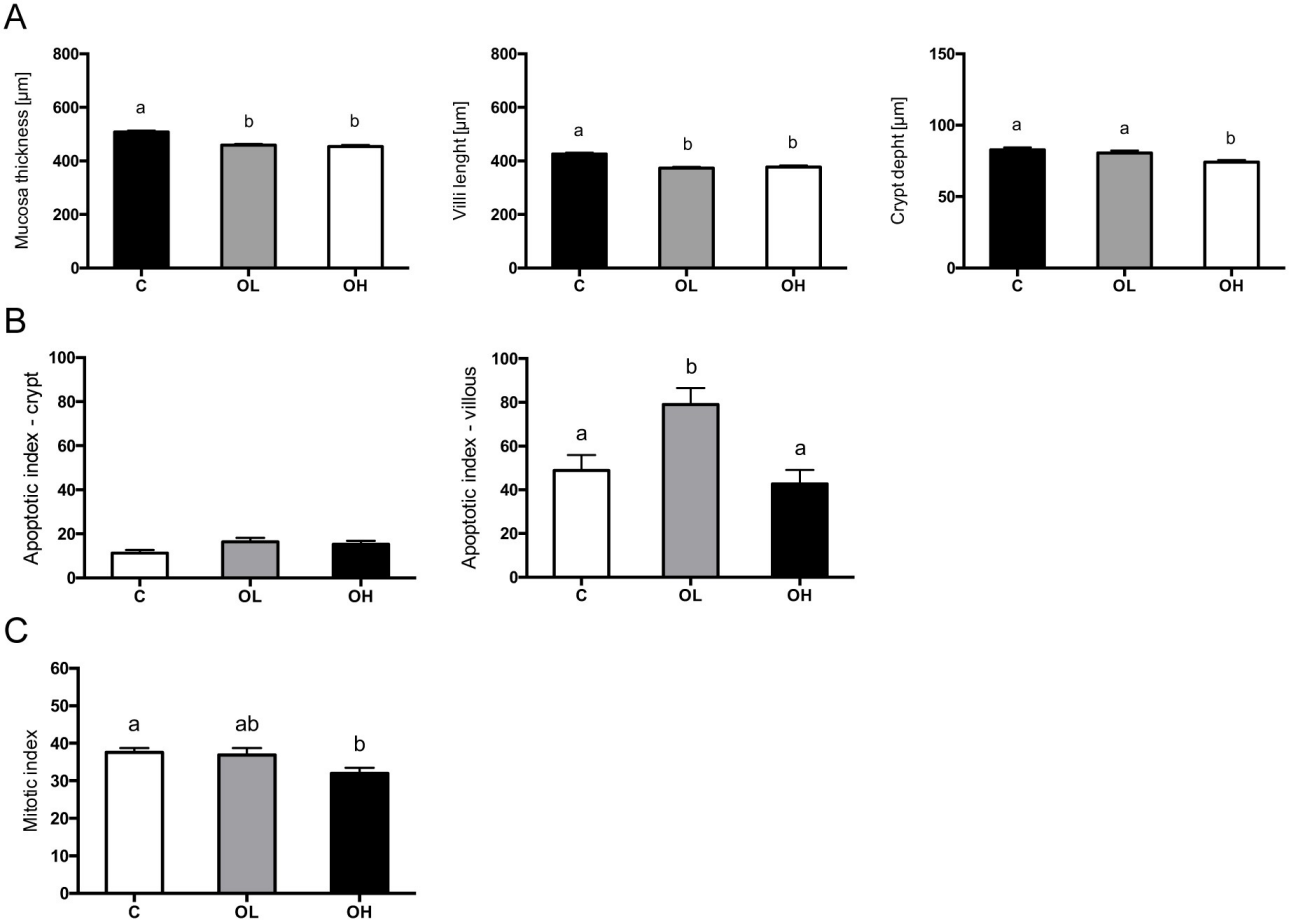
Obestatin effect on intestinal epithelium renewal in suckling rats. (A) The effect of enteral obestatin administration on histometry of small intestinal in suckling rats. (B) Apoptotic index (%) of crypt and villous small intestinal epithelial cells after enteral obestatin administration, n = 6 (C) Mitotic index (%) of crypt small intestinal epithelial cells after enteral administration of obestatin, n = 6. C—animals administered with saline solution (twice a day); OL—intra stomach obestatin (125 nmol/kg BW, twice a day); OH—intra stomach obestatin (250 nmol/kg BW, twice a day); values are given as means ± SEM; different superscript letters indicate statistical differences between the groups; p≤ 0.5.

**Fig 5:**
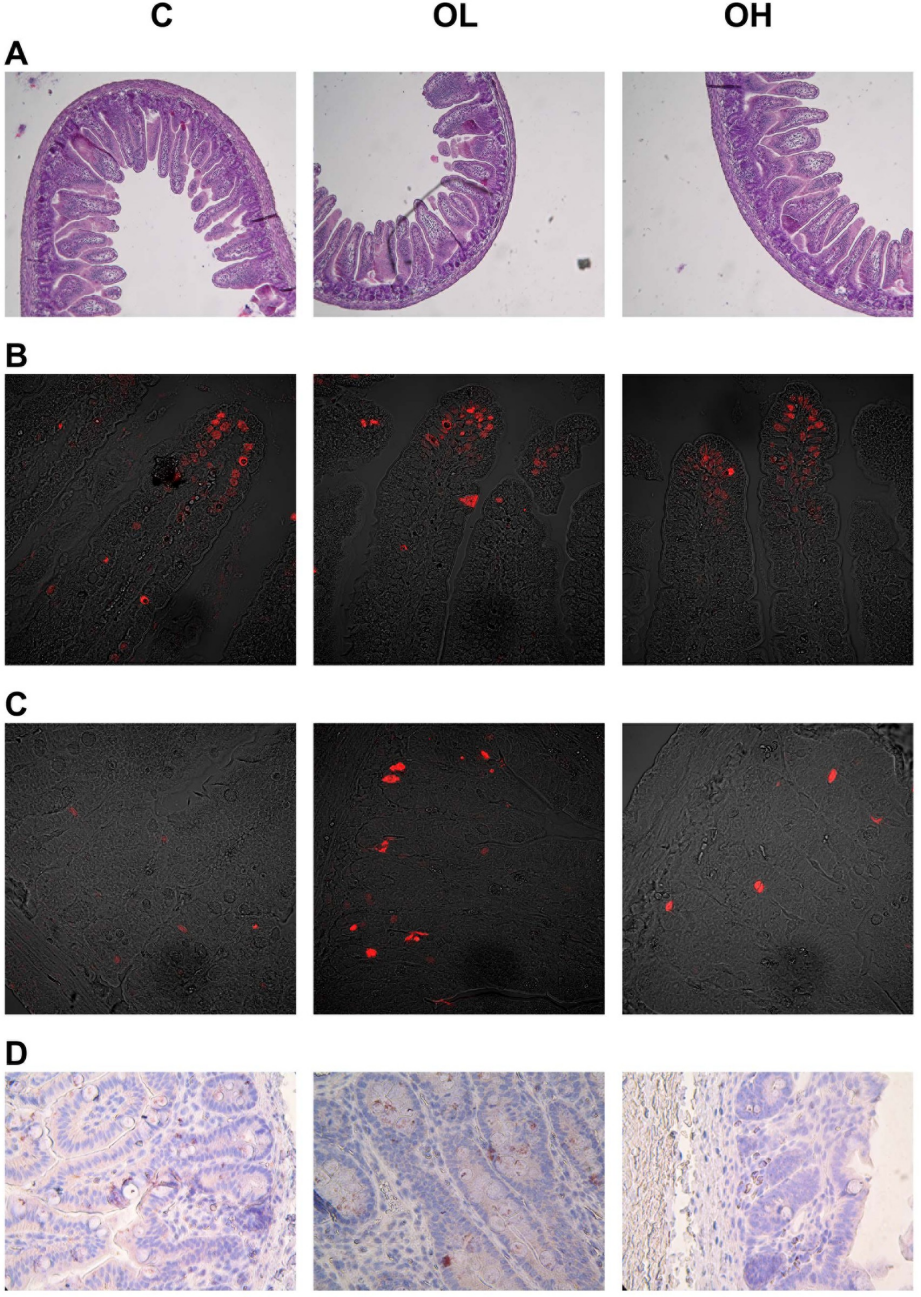
Microscopic images in representative cross-sections from the small intestine mucosa of suckling rats. (A) Histometry of small intestinal wall in suckling rats (light microscope, 100x). (B) Apoptosis of villous small intestinal epithelial cells (confocal microscope, 400x). (C) Apoptosis of crypt small intestinal epithelial cells (confocal microscope, 400x). (D) Mitosis of crypt small intestinal epithelial cells (light microscope, 400x). C—animals administered with saline solution (twice a day); OL—intra stomach obestatin (125 nmol/kg BW, twice a day); OH—intra stomach obestatin (250 nmol/kg BW, twice a day).

Enteral administration of both studied doses of obestatin resulted in significant decrease in the thickness of mucosa layer and length of the villi as compare to C group (p = 0.0001). Moreover, in the OH group also the depth of the intestinal crypts were significantly decreased (p = 0.0003) as compare to control animals (C), (Figs 4A and 5A). We observed, increase in the apoptosis rate (p = 0.0061) on the top of the villi in the group receiving lower dose of obestatin (OL) as compare to control group C, (Figs 4B and 5B). However, the apoptosis in the crypts were unaffected (Figs 4B and 5C). Administration of obestatin in higher dose decreased (p = 0.0228) proliferation activity of the crypt stem cells (Figs 4C and 5D).

### Obestatin effect on the activity of brush border enzymes

Administration of obestatin in a high dose (OH) had no statistically signicficant effect on the activity of examined brush border enzymes (lactase, aminopeptidase A and N, sucrose, maltase, dipeptidyl peptidase 4, sucrase) comparing to suckling animal treated only with saline solution (C). To distinguish, lower dose of obestatin administrated enterally changes the profile of enzymes activity observed in suckling rats. Compare with control animals (C) treating rats with 125 ug/kg BW for seven days (OL) resulted in elevation in lactase activity (p = 0.0265) and reduction in aminopeptidase N (p = 0.0339) and maltase (p = 0.0220) (Table 3).

**Table 3:** The activity of brush border enzymes after enteral administration of obestatin to suckling rats.

Parameter / Group	C	OL	OH	*P*
Protein (mg/ml)	500 ± 8^a^	465 ± 7^b^	456 ± 10^b^	*0*.*0022*
Aminopeptidase A	2.68 ± 0.10	2.35 ± 0.17	2.89 ± 0.24	*0*.*3820*
Aminopeptidase N	1.65 ± 0.12^a^	1.21 ± 0.13^b^	1.65 ± 0.15^ab^	*0*.*0339*
Dipeptidase IV	0.98 ± 0.05	0.92 ± 0.07	0.95 ± 0.08	*0*.*7827*
Lactase	1.39 ± 0.20^a^	2.34 ± 0.29^b^	1.81 ± 0.11^ab^	*0*.*0294*
Maltase	0.12 ± 0.01^a^	0.07 ± 0.01^b^	0.12 ± 0.009^a^	*0*.*0220*
Sucrase	0.62 ± 0.03^ab^	0.92 ± 0.10^a^	0.52 ± 0.06^b^	*0*.*0265*

C—saline solution (twice a day); OL—intra stomach obestatin (125 nmol/kg BW, twice a day); OH—intra stomach obestatin (250 nmol/kg BW, twice a day); values are given as means ± SEM; different superscript letters indicate statistical differences between the groups; p≤ 0.05.

### DNA repair activity assay

**Fig 6:**
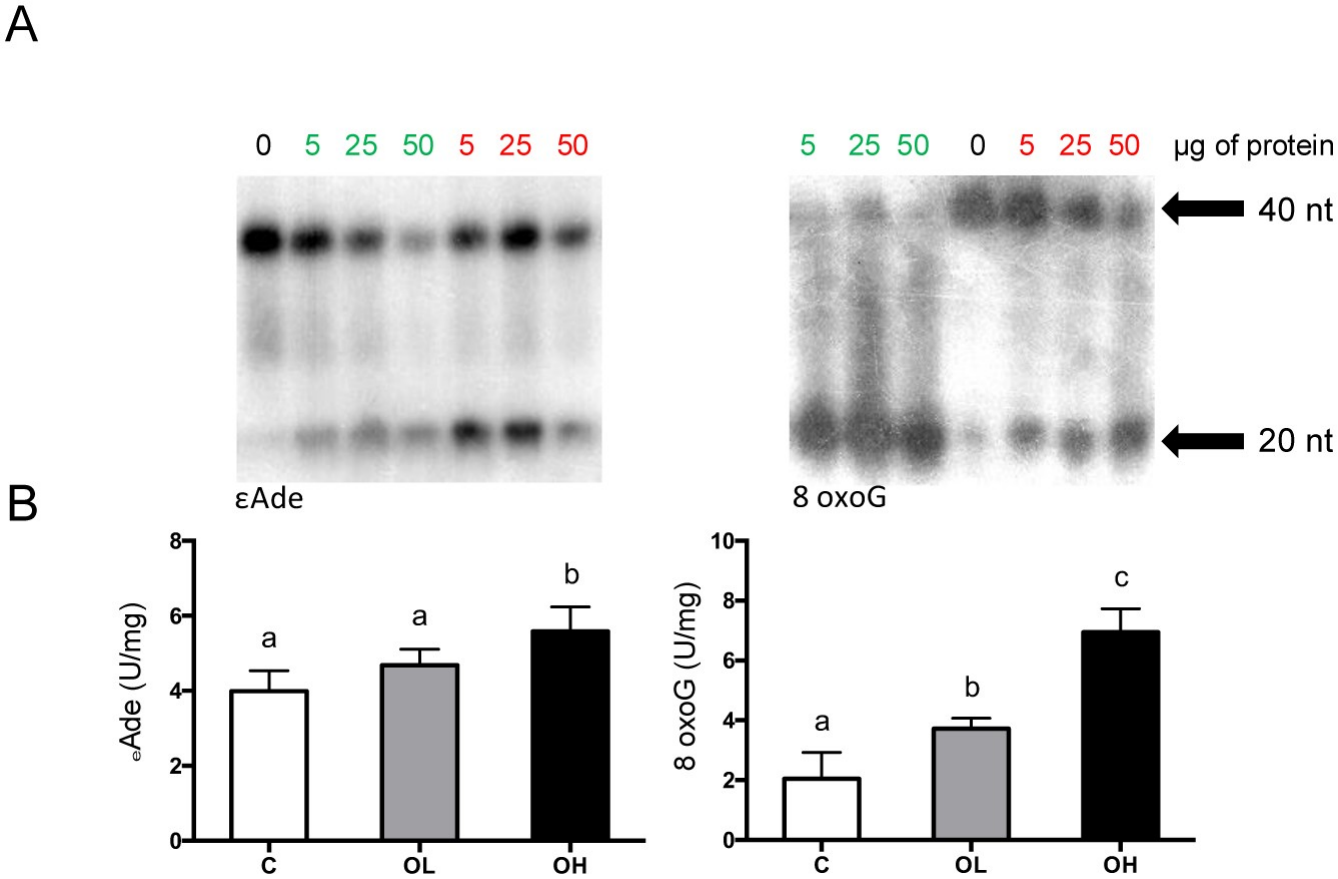
Obestatin effect on the excision of ethenoadducts in the small intestine of suckling rats. (A) ethenoadducts excision by increasing amounts of extracts from analyzed samples (green numbers) and control samples (red numbers). The amount of cleaved oligodeoxynucleotide was estimated by PhosphorImaging, and repair capacity was calculated from the linear part of the curve. The activity of 8oxo-G in the small intestinal segments. (B) the activity of eteno-A in the small intestine samples. C—animals administered with saline solution (twice a day); OL—intra stomach obestatin (125 nmol/kg BW, twice a day); OH—intra stomach obestatin (250 nmol/kg BW, twice a day); values are given as means ± SEM; different superscript letters indicate statistical differences between the groups; p≤ 0.05.

The repair activity assay revealed significant increase in 8oxo-G in both groups supplemented with obestatin (p< 0.001) in comparision to C group. Moreover, the observed increase was dose dependent. The rate of eteno-A in the jejunum was significantly elevated (p = 0.0021) in rats administered with high dose of obestatin as compare to other studied groups (Fig 6).

### Obestatin effect on Cox-2 expression

**Fig 7:**
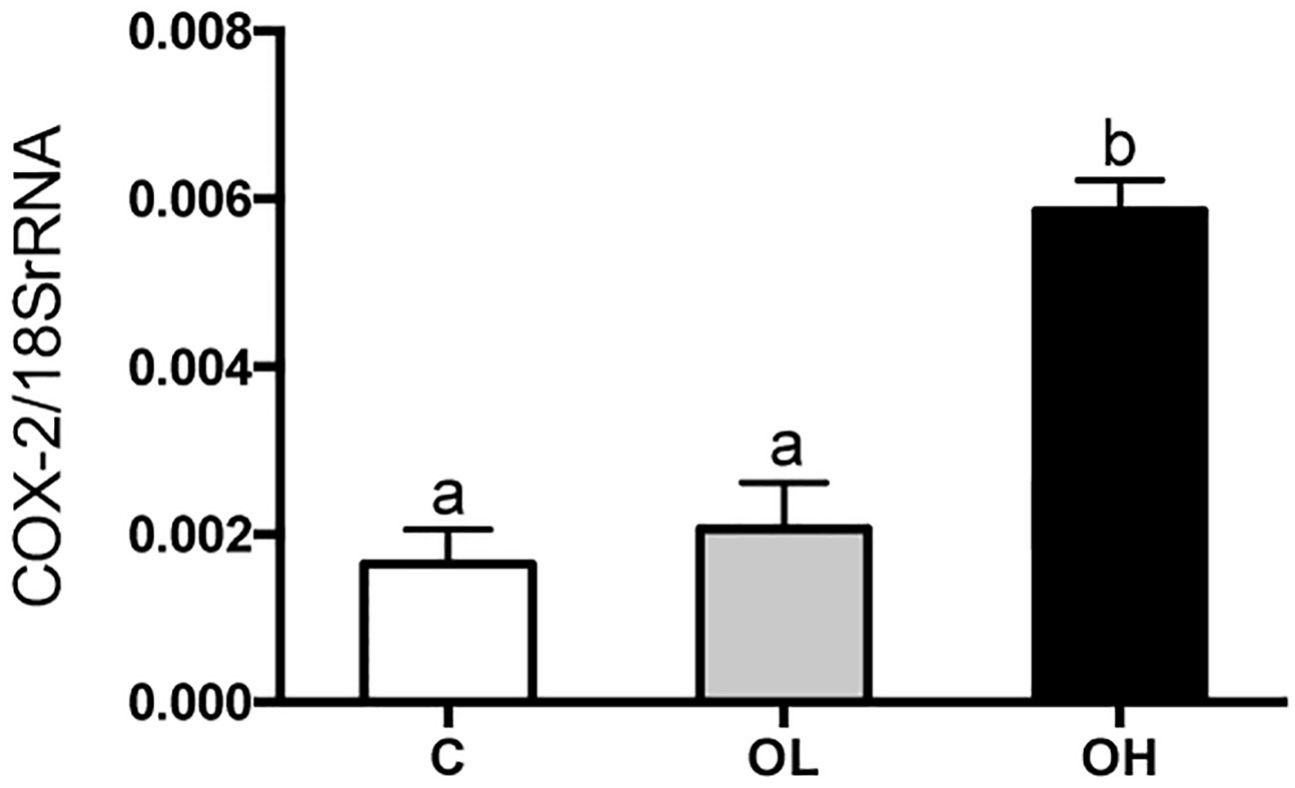
The level of COX-2 mRNA in relation to 18 S rRNA in extracts from rats small intestine. C—animals administered with saline solution (twice a day); OL—intra stomach obestatin (125 nmol/kg BW, twice a day); OH—intra stomach obestatin (250 nmol/kg BW, twice a day); values are given as means ± SEM; different superscript letters indicate statistical differences between the groups; p ≤ 0.05.

The expression of COX-2 mRNA was significantly increased (p< 0.0001) in the group administered with the high dose of obestatin (OH) as compared to group supplemented with lower dose of obestatin (OL) and control animals (C), (Fig 7).

## Discussion

Previous studies of our team indicated that obestatin when administered enterally in pharmacological doses 125 nmol/kg BW or 250 nmol/kg BW significantly influences small intestinal contractility of suckling rat neonates by decreasing the amplitude of contraction. The observed obestatin effect was independent both from Cajal cells and expression of M2 muscarinic receptors [4]. The above mentioned study together with previous studies of our team showing ghrelin impact on remodeling of the small intestinal mucosa in neonatal rats [10] were a premise to studies on obestatin effect on the process of intestinal development in suckling rats. As the process of development consists of both growth and maturation we presented comprehensive study on both structural growth and changes in the activity of brush border enzymes, which provide functional gut maturation. In the present study we shown that enteral administration of obestatin when given in dose 250 nmol/kg b.wt twice a day to suckling rats significantly increase the level of obestatin in the circulation but markedly inhibits endogenous synthesis of obestatin in the stomach what was confirmed both by immunocytochemical analysis showing the percentage of cells containing visible antigen irrespectively of its amount, and by Western blotting that shows total amount of antigen in tissue homogenates. The administration of lower dose of obestatin (125 nmol/ kg BW) had no significant effect on the obestatin concentration in the blood but significanty increased the endogenous expression of the peptide what was confirmed by Western blotting.

In both doses, enteral administration of obestatin effects on the intestinal development. In case of treatment with lower dose of obestatin we observed increase in the lactase activity together with the significant decrease in the activity of maltase what may suggest the retardment in the intestinal maturation. In comparison to control rat pups suckling their mothers we observed that administration of lower dose of obestatin decreases villi length due to increased apoptosis of small intestinal epithelial cells. Unlike lower dose, obestatin administered in dose 250 nmol/kg BW had no effect on the activity of brush border enzymes. However we observed changes in the structure of intestinal epithelium (decreased length of the villi and crypt depth) resulting from significant decrease in stem cell proliferation. These results are surprising as previous studies on colonic mucosa under colitis, human retinal epithelial cells [22] and porcine preadipocites [23] revealed that obestatin enhances proliferation and inhibits apoptosis. The reason of these differences is hard to explain however two aspects should be underline. First, in our study we used higher doses of obestatin than abovementioned studies. Moreover, our previous studies have shown that obestatin effect is strongly dependent on the age of animals and in consequence influenced by the number of myenteric neurons, functionally active receptors and their sensitivity, as well as the release of neurotransmitters, thus in turn affecting obestatin induced contractility.

As the higher dose of obestatin administered enterally managed to inhibit significantly the expression of endogenous obestatin in the stomach we performed micrroarays to check the consequences of this silencing. We showed that higher dose of obestatin effects on the profile of 131 genes. These genes among others are involved in neutrophil recruitment, priming and activation. The anti-inflammatory effect of obestatin regulated by the neutrophils were previously reported. However, in our study we have also observed the increase in the rate of 8-oxo G and eteno A together with the increased COX-2 expression. Etheno-DNA adducts are mutagenic and carcinogenic and they are formed by the reaction of lipidperoxidation (LPO) products as well as being induced by external environmental factors stimulated by a specific diet. These factors induce the formation of alkylation of damaged bases in etheno-DNA, which includes 1,N^6^-etheno-2′-deoxyadenosine (εdA), 3,N^4^-etheno-2′-deoxycytidine (εdC), 1,N^2^-etheno-2′-deoxyguanosine (1,N2-εdG) and the oxidation of bases whose marker in the cell is 8-oxo-7,8-dihydroguanine, (8oxoG), [24]. These lesions are recognized and repaired by enzymes from the repair glycosylation group: ANPG for (εdA), and OGG1 for (8oxoG), in the base excision repair (BER) process [24,25]. One of the reasons the LPO products are generated is inflammation driven by oxidative stress. Oxidative stress is a complication that occurs when the generation of reactive oxygen species (ROS) exceeds antioxidant enzyme activity [26]. Moreover, we have observed increased expression of Cox-2 which posses pro-inflammatory activities and is up-regulated by hormones, growth factors but also during inflammation. Cox-2 is synthetized by active neutrophils what leads to the synthesis of prostaglandin (PE) E2, an important mediator of many biological functions, such as regulation of immune responses, blood pressure, gastrointestinal integrity, and fertility [27]. All together suggests that obestatin when given enterally in dose 250 nmol/kg BW activates signaling pathway connected with inflammation and the integrity of the small intestinal mucosa. Opposite effect of obestatin was shown in several studies claiming anti-inflammatory [28], anti-oxidant and anti-apoptotic properties [29]. The observed opposite effects of obestatin may be connected with aspects which were mentioned above like the age related changes in the density of obestatin receptors or/and the route of peptide administration. It should be highlighted that studies showing anti-inflammatory, anti-oxidant and anti-apoptotic effects of obestatin investigated obestatin role in disease not in the physiological state. Pomukcu et al [28] shown that obestatin has anti-inflammatory effect in rat colitis but the effectiveness of this effect was dependent on its form (acute *vs*. chronic). That suggests that state of immune system determine the response of obestatin. It should be also mentioned that gene profile revealed that key regulated genes after obestatin administration in higher dose are mitogen-activated protein kinases: MAPK3 and MAP2K3 which regulate pathways mediating inflammation, cell survival, cell death, proliferation and differentiation. It should be mentioned that obestatin intracellular mechanism of action via MAP kinase and p53 was previously reported in the studies on porcine ovarian granulosa cells [30]. Our observations provide evidence that enteral obestatin besides influencing intestinal contractility effects small epithelial cell remodeling in suckling rats. The profile of changes observed in the small intestine after enteral treatment with obestatin suggests that this effect in case of long lasting administration will not be supportive for growth and development but could be even harmful. The studies showing lower concentration of obestatin in the maternal milk for preterm neonates as compare to milk of neonate born in time may support this observation. Maternal milk, the gold standard providing the best growth and development has limited amounts of obestatin for preterms susceptible for inflammation. The further studies are needed to understand obestatin role in the early postnatal development.
